# Low *EVI1* expression at diagnosis identifies a high-risk subgroup in adult Ph-negative B-cell acute lymphoblastic leukemia

**DOI:** 10.3389/fmed.2025.1701539

**Published:** 2026-01-13

**Authors:** Shu Kong, Xu Wang, Wen-Min Chen, Ling-Di Li, Yue Hao, Jin-Ying Li, Dai-Hong Xie, Zhao-Yu Li, Yue-Yun Lai, Hao Jiang, Qian Jiang, Ya-Zhen Qin

**Affiliations:** Peking University People’s Hospital, Peking University Institute of Hematology, National Clinical Research Center for Hematologic Disease, Beijing, China

**Keywords:** B-cell acute lymphoblastic leukemia, ecotropic viral integration site 1, fusion gene, prognosis, real-time quantitative RT-PCR

## Abstract

**Background:**

The prognostic impact of *EVI1* expression in B-cell acute lymphoblastic leukemia (B-ALL) remains to be explored.

**Method:**

Bone marrow (BM) samples collected from 436 consecutive newly diagnosed adult Ph-negative B-ALL patients were tested for *EVI1* transcript levels using real-time quantitative PCR.

**Result:**

The median *EVI1* transcript level in the whole cohort was 0.40% (range: 0.0030–94.3%). Low *EVI1* expression defined by lower three quartiles (*EVI1* transcript levels was 2.3%) was significantly related to poorer relapse-free survival (RFS) and overall survival (OS) (*p* = 0.0010 and < 0.0001) and was an independent adverse prognostic factor for RFS (HR (95% confidence interval): 2.3 (1.2–4.1), *p* = 0.0070) and OS (HR (95% CI): 2.5 (1.3–5.1), *p* = 0.0090) in the whole cohort. The optimal thresholds for *EVI1* transcript levels in the fusion gene subgroups were determined individually, and low *EVI1* expression was related to or tended to be related to poorer RFS in patients with *TCF3::PBX1*, *Ph-like* fusions, *MEF2D* fusions, and *ZNF384* fusions groups (*p* = 0.0047, 0.025, 0.032, and 0.070) and was associated with poorer OS in *ZNF384* fusions groups (*p* = 0.012), respectively. Furthermore, patients with *TCF3::PBX1* or *MEF2D* fusion and high *EVI1* expression had RFS and OS similar to those without the corresponding fusions, and patients with *ZNF384* fusion and low *EVI1* expression had RFS and OS comparable to those without *ZNF384* fusions (all *p* > 0.05).

**Conclusion:**

Low *EVI1* transcript levels at diagnosis are related to poor prognosis in adult Ph-negative B-ALL, and *EVI1* expression may improve fusion gene-defined risk stratification.

## Introduction

The ecotropic viral integration site 1 (*EVI1*) is a regulatory transcription factor that plays an important role in hematopoiesis and self-renewal (1). It was first identified as a common ecotropic viral integration in the DNA of AKXD murine myeloid tumors (2). In human malignancies, *EVI1* is frequently activated by chromosomal rearrangements at the 3q26.2 locus in acute myeloid leukemia (AML), myelodysplastic syndrome (MDS), and chronic myeloid leukemia (CML) (3, 4). The presence of 3q26.2 abnormalities in these diseases is consistently associated with adverse clinical outcomes (5–8). Notably, elevated *EVI1* expression is also common in AML patients without 3q26.2 abnormalities and similarly confers an adverse prognosis (9). Previously, our center had reported that high *EVI1* expression independently predicted poor outcomes in AML patients with intermediate cytogenetic risk (ICR-AML) receiving chemotherapy (10).

Compared with the extensive research on the expression and functional role of *EVI1* in myeloid malignancies, fewer studies have shown examined its involvement in lymphoid malignancies. Unlike in AML, 3q26.2 rearrangements are fairly rare in acute lymphoblastic leukemia (ALL) and have been reported only in isolated cases of secondary ALL (11, 12). Nevertheless, dysregulated *EVI1* expression has been observed in both pediatric and adult ALL (13–16). Konantz et al. have reported that *EVI1* expression contributes to the leukemogenic potential and apoptosis resistance of ALL cells (17). However, our group has reported that low *EVI1* expression was an independent adverse prognostic factor for relapse-free survival (RFS) and overall survival (OS) in pediatric B-ALL patients (18). In adult ALL, only two studies have been reported to date: one found no prognostic impact of either low or high *EVI1* expression on the cumulative incidence of failure (CIF, failure meaning primary refractoriness or relapse) and event-free survival (EFS) in Ph-negative B-cell ALL (B-ALL) patients, while the other showed no significant impact of *EVI1* expression levels on OS and disease-free survival (DFS) rates in ALL patients (including B- and T-ALL) (14, 16). With the recent identification of several new fusion genes that have refined molecular classification and risk stratification in B-ALL (19, 20), a comprehensive evaluation of the relationship between *EVI1* expression patterns, molecular subtypes, and prognostic significance remains a significant unmet need.

In this study, we conducted a large-scale cohort study enrolling 436 consecutive adult Ph-negative B-ALL patients. All patients underwent comprehensive screening for both classical and new fusion genes. We retrospectively performed real-time quantitative RT-PCR (RQ-PCR) to detect *EVI1* transcript levels in the bone marrow samples collected at diagnosis. Subsequently, we investigated the *EVI1* expression pattern and the prognostic significance.

## Patients and methods

### Patients and treatment

A total of 436 newly diagnosed adult Ph-negative B-ALL patients were included in this study. They were consecutively diagnosed from December 2009 to April 2024 and received at least one course of induction chemotherapy in our institute. There were 205 (47.0%) male patients in total. The median age at diagnosis was 33 (range 16 to 65) years. The diagnosis was based on bone marrow morphology, immunophenotyping, chromosomal karyotyping, and molecular testing. The last follow-up was conducted in September 2024.

The chemotherapy procedures in our institute have been reported in detail in previous studies (21, 22). Induction therapy was performed using the CODP±L (cyclophosphamide and prednisone or dexamethasone, vincristine, and daunorubicin or idarubicin and L-asparaginase) regimen. Patients who achieved first complete remission (CR1) subsequently received post-remission therapy, which consisted of either hyper-CVAD-based (high-dose methotrexate and cytarabine therapy) chemotherapy only or chemotherapy followed by allogeneic hematopoietic stem cell transplantation (allo-HSCT). The indications for allo-HSCT, pretreatment protocols, donor selection, and prevention of graft-versus-host disease have been described previously (23). Minimal residual disease (MRD) was assessed in all patients by multiparameter flow cytometry after each cycle of chemotherapy and after HSCT.

### Extraction of RNA and DNA

Bone marrow samples were collected from all patients at diagnosis for RNA and DNA extraction. Nucleated cells were obtained by treating fresh bone marrow samples with 0.144 M NH4Cl, 0.01 M NH4HCO3 to lyse the red cells. Total RNA and genomic DNA were individually extracted using Trizol and DNAzol reagents (Invitrogen, Carlsbad, CA, USA).

### Detection of classical and new fusion genes and *IKZF1* deletion

RNA was reverse transcribed into cDNA. TaqMan-based RQ-PCR was performed using cDNA to screen B-ALL-related classical fusion transcripts at diagnosis, including *BCR::ABL1*, *TCF3::PBX1*, *TEL::AML1*, and *KMT2A* rearrangement (*KMT2A::AFF1*, *KMT2A-MLLT1*, *KMT2A::MLLT6*, *KMT2A::EPS15*, and *KMT2A::MLLT11*. *IKZF1*) deletions were detected by RQ-PCR using DNA in 239 patients (24). Furthermore, all patients were retrospectively screened for new fusion transcripts, including *ZNF384*, *MEF2D*, and *Ph-like* fusion genes using TaqMan-based multiplex RQ-PCR, as previously reported (25).

### Detection of *EVI1* transcript levels

As reported in our previous study (10), TaqMan-based RQ-PCR was used to measure *EVI1* transcript levels. The primers and probes for the *EVI1* transcript were designed using Primer Express Software (Applied Biosystems, Foster City, USA) to detect all subtypes (1a, 1b, 1c, 1d, and 3 L), and the sequences were as follows:

Forward primer: 5’-CCCATGTGCCAGAGGAACTT-3′ (in exon 14).

Reverse primer: 5’-CAGTGACAGCATCATAGCATATGC-3′ (in exon 15).

Probe: 5’-FAM-CAGCCGTTACACAGAAAGTCCAAATCGC-TAMRA-3′ (in exon 14).

The primers and probe for *ABL1* were based on a report from the Europe Against Cancer Program (26). The amplification efficiency of *EVI1* and *ABL1* was validated by constructing standard curves, both of which fell within the acceptable range of 90–110% with R^2^ > 0.99 (Supplementary Figure S1). The *EVI1* transcript level was calculated as *EVI1* copies /*ABL1* copies in percentage.

### Definitions and statistical analysis

Complete remission (CR) was defined as the absence of circulating lymphoblasts and extramedullary disease, restoration of trilineage hematopoiesis (TLH) and <5% leukemic blasts, a neutrophil count of more than 1 × 10^9^/L, and a platelet count of more than 100 × 10^9^/L (20). Relapse was defined as the recurrence of more than 5% of BM blasts, reappearance of peripheral blasts in the blood, or in any extramedullary site after CR. Relapse-free survival (RFS) was measured from the date of achieving CR to relapse or to the last bone marrow testing. Overall survival (OS) was measured from the date of diagnosis to the date of death (regardless of cause) or the last follow-up. High-risk karyotype was defined based on the Eastern Cooperative Oncology Group (ECOG) 2,993 trial (27). Fusion transcript testing results by RQ-PCR were also considered.

The Mann–Whitney *U* test was performed on continuous variables, and the chi-squared or Fisher’s exact test was performed on categorical variables. The optimal cutoff levels were determined by dividing *EVI1* transcript levels into quartiles or using receiver operating characteristic (ROC) curves based on different patient responses (achieving CR). Survival functions were estimated using the Kaplan–Meier method and were compared using the log-rank test. Variables with a *p-*value of < 0.05 from the univariate analyses were entered into the multivariate model using Cox proportional hazards models to identify the most statistically significant parameters associated with RFS and OS. The level of statistically significant difference was set at a *p-*value of < 0.05. SPSS software 26.0 (IBM Corporation, Armonk, NY) and GraphPad Prism 10 (GraphPad Software Inc., La Jolla, CA) were used for statistical analysis.

## Results

### Patient outcomes

In the entire cohort, 405 (92.9%) patients achieved CR after induction chemotherapy, and 136 patients subsequently relapsed with a median time of 5.5 (range: 0.67–69.5) months. Of the 405 patients who achieved CR, 148 received chemotherapy alone, while the remaining 257 patients underwent allo-HSCT after chemotherapy. The median follow-up duration for all 436 patients was 25.7 (range: 1.0–155.7) months. A total of 291 (66.7%) patients were alive at the last follow-up with a median follow-up time of 41.0 (range: 1.0–155.7) months. The 4-year RFS and OS rates of the entire cohort were 64.0% (95% confidence interval (CI): 58.5–68.9%) and 63.7% (95% CI: 58.4–68.5%), respectively.

### Molecularly and cytogenetically defined patient groups

In the entire cohort, 186 (42.7%) patients were identified with fusion genes (Supplementary Table S1). A total of 23 (5.3%) patients had the *TCF3::PBX1* fusion transcripts; 39 (8.9%) had an *KMT2A* rearrangement, including *KMT2A::AFF1* (*n* = 35), *KMT2A::MLLT1* (*n* = 3) and *KMT2A::EPS15* (*n* = 1); 73 (16.7%) had *ZNF384* fusion transcripts, including *EP300::ZNF384* (*n* = 56), *CREBBP::ZNF384* (*n* = 8), *TAF15::ZNF384* (*n* = 4), *TCF3::ZNF384* (*n* = 3), and *EWSR1::ZNF384* (*n* = 2); 21 (4.8%) had *MEF2D* fusion transcripts, including *MEF2D::BCL9* (*n* = 11), *MEF2D::HNRNPUL1* (*n* = 8), *MEF2D::DAZAP1* (*n* = 1), and *MEF2D::FOXJ2* (*n* = 1); 19 (4.3%) had *Ph-like* fusion transcripts, including *P2RY8::CRLF2* (*n* = 6), *EBF1::PDGFRB* (*n* = 3), *RCSD1::ABL2* (*n* = 3), *NUP214::ABL1* (*n* = 2), *TEL::ABL1* (*n* = 1), *EBF1::JAK2* (*n* = 1), *PCM1::JAK2* (*n* = 1), *PAX5::JAK2* (*n* = 1), and *BCR::FGFR1* (*n* = 1); and 11 (2.5%) had hyperdiploidy karyotype. The remaining patients (*n* = 250, 57.3%) were defined as B-other in the current study. It should be noted that neither chromosome 3q26.2 abnormalities nor cryptic 3q26.2 rearrangements were found in the whole cohort.

### *EVI1* expression patterns in patients at diagnosis

The median *EVI1* transcript levels for all patients were 0.40% (range:0.0030–94.3%). Compared with 27 normal bone marrow (NBM) samples collected from healthy donors, as we previously reported (10), Ph-negative B-ALL patients had significantly lower *EVI1* transcript levels (*p* < 0.0001).

**Figure 1:**
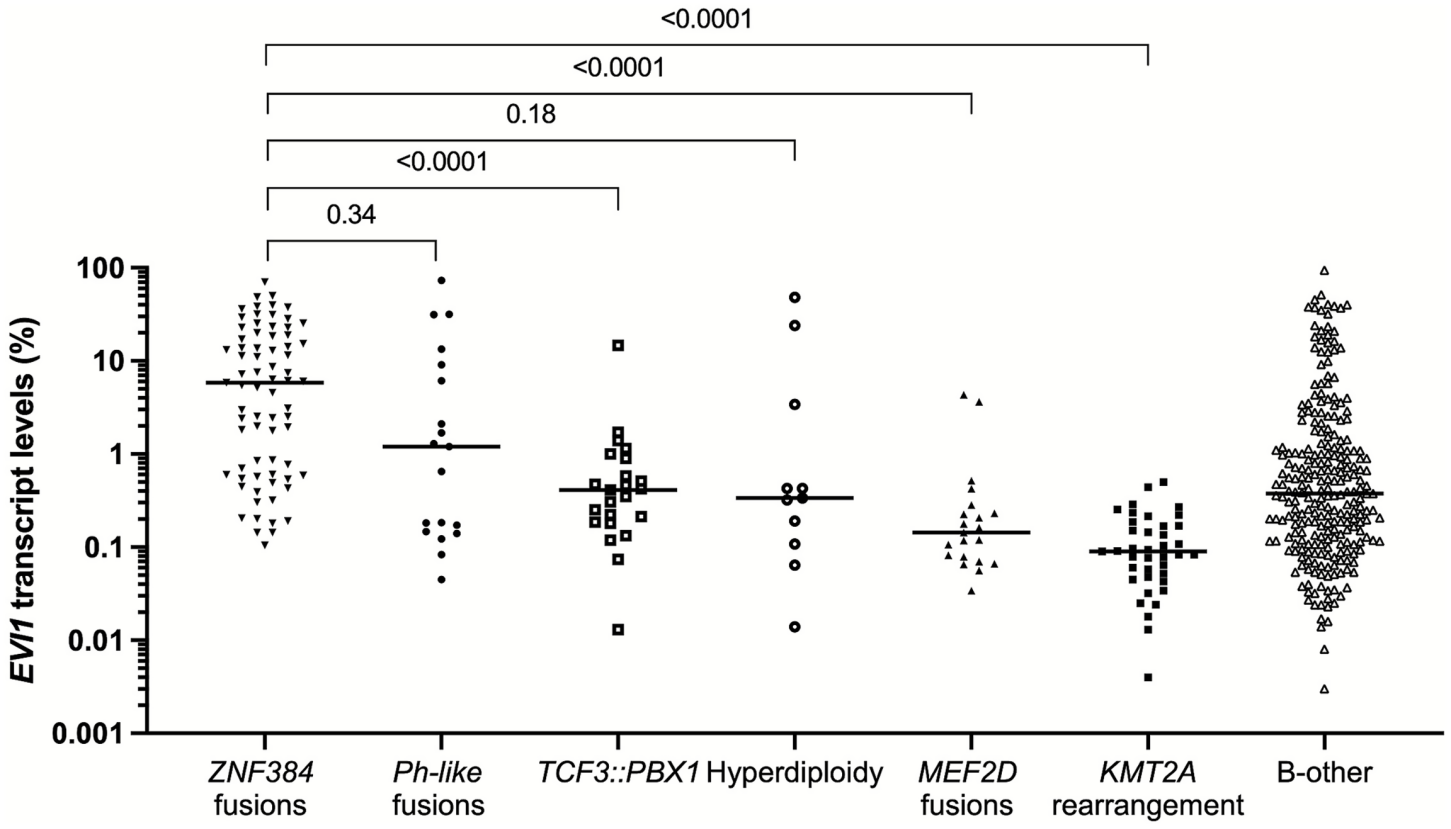
*EVI1* expression patterns of molecularly and cytogenetically defined B-ALL groups.

As shown in Figure 1 and Supplementary Table S1, the *EVI1* transcript levels were varied significantly among the *ZNF384* fusion, *Ph-like* fusion, *TCF3::PBX1* fusion, hyperdiploidy, *MEF2D* fusion, *KMT2A* rearrangement, and B-other groups (*p* < 0.0001) with the median levels of 5.8% (range: 0.11–70.6%), 1.2% (range: 0.045–72.9%), 0.41% (range: 0.013–14.7%), 0.34% (range: 0.014–48.2%), 0.14% (range: 0.034–4.3%), 0.090% (range: 0.0040–0.50%), and 0.38% (range: 0.003–94.3%), respectively. The *ZNF384* fusion group had significantly higher *EVI1* transcript levels than *TCF3::PBX1* fusion, *MEF2D* fusion, *KMT2A* rearrangement, and B-other groups (all *p* < 0.0001) and had no statistically significant difference compared to *Ph-like* fusion and hyperdiploidy groups (*p* = 0.34 and 0.18).

### Determining the optimal cutoff value of the *EVI1* transcript levels for patient grouping

**Figure 2:**
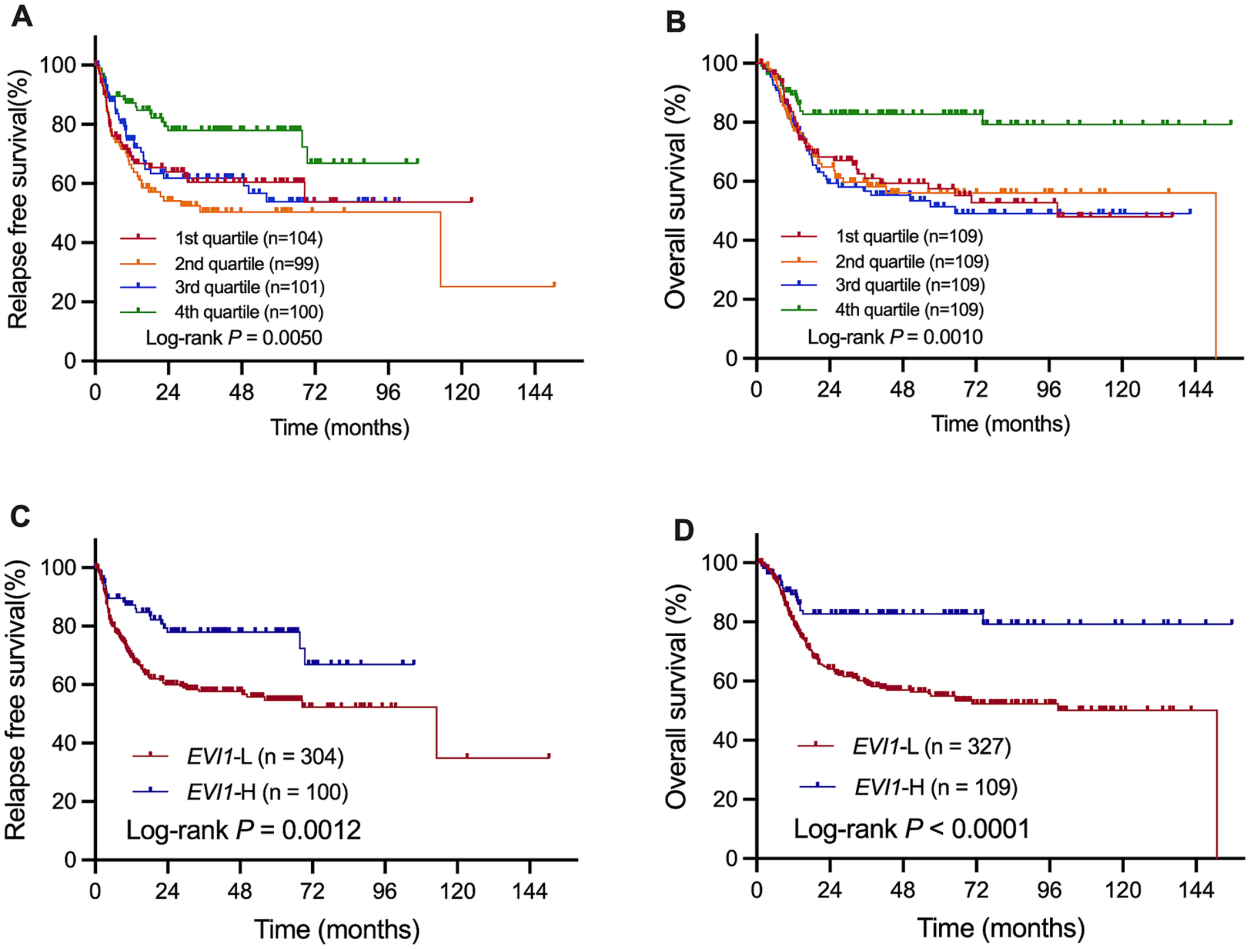
RFS and OS analysis in the entire cohort. Patients were grouped into quartiles according to *EVI1* transcript levels **(A,B)**; comparison of RFS **(C)** and OS **(D)** between *EVI1*-L and *EVI1*-H group.

Patients were grouped into quartiles according to *EVI1* transcript levels (1st quartile to fourth quartile, from low to high levels). As shown in Figure 2, both RFS and OS were significantly different among the four groups (*p* = 0.0050 and *p* = 0.0010, Figures 2A,B). The bottom three quartile groups demonstrated significantly poorer RFS and OS than the top quartile group (RFS: *p* = 0.012, 0.0004, and 0.014, OS: *p* = 0.0010, 0.0060, and 0.0001, respectively), while no significant differences were observed among the remaining three groups (RFS: *p* = 0.37, OS: *p* = 0.82). Therefore, we used the upper quartile (top 25%, *EVI1* transcript levels were 2.3%) as the cutoff value to stratify the patients into *EVI1*-H (*n* = 109) and *EVI1*-L (*n* = 327) groups.

### Relationship between *EVI1* expression and patient characteristics

As shown in Table 1, in the entire cohort, low *EVI1* expression was significantly related to *KMT2A* rearrangement, *TCF3::PBX1* fusion, *ZNF384* fusion, *IKZF1* deletion, high-risk karyotype, and fusion-defined high-risk group (*p* < 0.0001, 0.019, < 0.0001, 0.036, 0.0006, and < 0.0001), respectively. A non-significant trend was also observed for an association with lower platelet counts (*p* = 0.051). In contrast, *EVI1* expression had no relationship with age, sex, WBC counts, hemoglobin, hyperdiploidy karyotype, *MEF2D* fusion, *Ph-Like* fusion, and immunophenotype-defined group (all *p* > 0.05).

**Table 1:** Relationship between *EVI1* expression and variables at diagnosis in adult Ph-negative B-ALL.

Variable	All	*EVI1* transcript levels		*p* value*
		*EVI1*-L	*EVI1*-H	
*N*	436	327	109	–
Age (y; median; range)	33 (16–65)	33 (16–65)	31 (16–63)	0.36
Male/Female	205/231	151/176	54/55	0.54
WBC counts (×10^9^/L; median; range)	8.7 (0.3–535.2)	8.8 (0.3–535.2)	8.2 (0.7–249.4)	0.41
Hemoglobin (g/L; median; range)	87.0 (28.0–165.0)	87.0 (28.0–165.0)	87.0 (40.2–154.0)	0.58
Platelet counts (×10^9^/L; median; range)	67.0 (0.1–510.0)	54.5 (0.1–510.0)	112.0 (11–368.0)	0.051
Hyperdiploidy Karyotype (*n* = 335)				0.90
Yes	11	8	3	
No	324	241	83	
*KMT2A* rearrangement/t(4;11)				< 0.0001
Negative	397	288	109	
Positive	39	39	0	
*TCF3::PBX1*/t(1;19)				0.019
Negative	413	305	108	
Positive	23	22	1	
*ZNF384* fusion				< 0.0001
Negative	363	300	63	
Positive	73	27	46	
*MEF2D* fusion				0.093
Negative	415	308	107	
Positive	21	19	2	
*Ph-like* fusion				0.50
Negative	417	314	103	
Positive	19	13	6	
*IKZF1* deletion (*n* = 239)				0.036
Negative	189	145	44	
Positive	50	31	19	
Cytogenetic categories (*n* = 335)**				0.0060
High-risk karyotype	55	49	6	
Non-high-risk karyotype	280	200	80	
Immunophenotyping (*n* = 370)				0.52
Common-B	235	177	58	
Pre-B	55	45	10	
Pro-B	80	59	21	
Fusion-defined group^#^				< 0.0001
Standard-risk	334	234	100	
High-risk	102	93	9	

*The *p* values presented a comparison between the EVI1-L and EVI1-H groups using the chi-squared or Fisher’s exact test.

**Based on the ECOG 2993 trial [ref (27)], fusion transcript testing results by RQ-PCR were also considered.

^#^Based on previous research published by our center and an international study of 1,223 cases [ref (28, 37)].

### The impact of *EVI1* expression at diagnosis on CR achievement

In the whole cohort, the CR rate after one course of induction therapy in the *EVI1*-L group was significantly higher than that in the *EVI1*-H group (88.1% versus 78.9%, *p* = 0.018); while the CR rate after two courses of induction therapy wase similar between them (90.4% versus 89.7%, *p* = 0.84).

### Low *EVI1* expression predicted poor outcome in the whole cohort

As shown in Figure 2, both RFS and OS were significantly poorer in the *EVI1*-L group than those in the *EVI1*-H group in the entire cohort (4-year RFS rate: 57.9% [95% confidence interval (CI) 51.2–63.6%] vs. 78.0% [95% CI 67.5–85.4%], *p* = 0.0012, Figure 2C; 4-year OS rate: 57.0% [95% CI 50.7–62.8%] vs. 82.7% [95% CI 73.6–88.9%], *p* < 0.0001, Figure 2D). In the chemotherapy only subgroup, *EVI1*-L patients showed a trend of poorer OS than *EVI1*-H patients, but no significant differences was observed in RFS between them (*p* = 0.092 and 0.40, Supplementary Figures S2A,B); while in allo-HSCT subgroup, the *EVI1*-L patients had significantly lower RFS and OS rate than *EVI1*-H patients (*p* = 0.026 and 0.0003, immunophenotyping defined group Supplementary Figures S2C,D).

### Univariate and multivariate analyses of RFS and OS in the whole cohort

**Figure 3:**
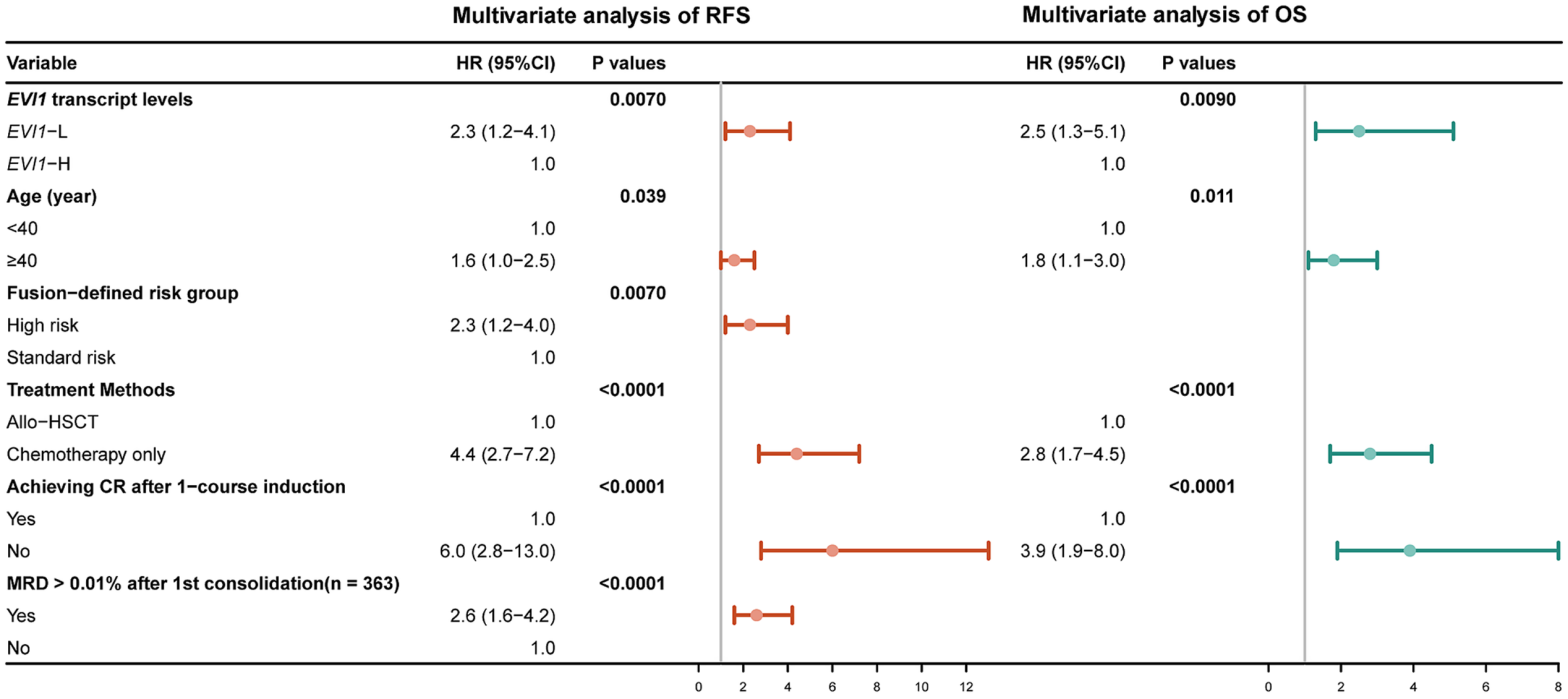
Forest plots for multivariate analysis of RFS and OS.

In the entire cohort, except for low *EVI1* transcript levels, age ≥ 40 years, WBC ≥ 20×10^9^/L, platelet count < 67×10^9^/L, high-risk karyotype, fusion-defined high-risk group (28), treated with chemotherapy only, no CR after one course induction, and MRD > 0.01% after the first consolidation were all significantly associated with a lower RFS rate (all *p* < 0.05, Table 2). As shown in Figure 3, the multivariate analysis showed that *EVI1*-L (HR (95%CI): 2.3 (1.2–4.1), *p* = 0.0070), age ≥ 40 years (HR (95%CI): 1.6 (1.0–2.5), *p* = 0.039), fusion-defined high-risk group (HR (95%CI): 2.3 (1.2–4.0), *p* = 0.0070), treating with chemotherapy only (HR (95%CI): 4.4 (2.7–7.2), *p* < 0.0001), no CR after one course induction (HR (95%CI): 6.0 (2.8–13.0), *p* < 0.0001), and MRD > 0.01% after the first consolidation (HR (95%CI): 2.6 (1.6–4.2), *p* < 0.0001) were independent poor prognostic factors for RFS.

**Table 2:** Univariate and multivariate analysis of RFS in the entire cohort.

Variable	Univariate analysis		Multivariate analysis	
	4-year RFS Rate (95%CI)	*p* values*	HR (95%CI)	*p* values
*EVI1* transcript levels		0.0010		0.0070
*EVI1*-L	57.9% (51.2–63.6%)		2.3 (1.2–4.1)	
*EVI1*-H	78.0% (67.5–85.4%)		1.0	
Age (year)		0.004		0.039
<40	67.0% (60.3–72.8%)		1.0	
≥40	54.5% (44.8–63.3%)		1.6 (1.0–2.5)	
Sex		0.74		
Male	62.4% (54.3–69.5%)			
Female	63.3% (55.6–70.0%)			
WBC (x10^9^/L)		0.011		
<20	66.1% (59.6–71.8%)			
≥20	54.7% (44.1–64.1%)			
Hb (g/L)		0.72		
<87	63.3% (55.4–70.2%)			
≥87	62.3% (54.5–69.2%)			
Plt (x10^9^/L)		< 0.0001		
<67	53.0% (44.9–60.3%)			
≥67	72.6% (65.1–78.7%)			
Cytogenetic categories (*n* = 335)		< 0.0001		
High-risk karyotype	50.8% (35.2–64.5%)			
Non-high-risk karyotype	70.3% (63.5–76.0%)			
*IKZF1* deletion		0.53		
Positive	64.0% (46.5–77.1%)			
Negative	71.6% (63.3–78.3%)			
Fusion-defined risk group		< 0.0001		0.0070
High risk	44.2% (32.5–55.3%)		2.3 (1.2–4.0)	
Standard risk	68.3% (62.1–73.7%)		1.0	
Treatment methods		< 0.0001		<0.0001
Allo-HSCT	79.8% (73.5–84.7%)		1.0	
Chemotherapy only	29.5% (21.3–38.1%)		4.4 (2.7–7.2)	
Achieving CR after 1-course induction		< 0.0001		<0.0001
Yes	65.0% (59.3–70.0%)		1.0	
No	34.5% (14.8–55.5%)		6.0 (2.8–13.0)	
MRD > 0.01% after first consolidation (*n* = 363)		0.0010		<0.0001
Yes	54.5% (44.8–63.3%)		2.6 (1.6–4.2)	
No	68.4% (61.3–74.6%)		1.0	

*Variables with *p* < 0.05 from the univariate analyses were entered into the multivariate model using Cox proportional hazards models.

As shown in Table 3, for OS, in addition to low *EVI1* expression, age ≥ 40 years, WBC ≥ 20×10^9^/L, platelet count < 67×10^9^/L, high-risk karyotype, fusion-defined high-risk group, treated with chemotherapy only, and no CR after one course of induction were all associated with a lower OS rate (all *p* < 0.05). The multivariate analysis revealed that low *EVI1* expression (HR (95%CI): 2.5 (1.3–5.1), *p* = 0.0090), age ≥ 40 years (HR (95%CI): 1.8 (1.1–3.0), *p* = 0.011), treating with chemotherapy only (HR (95%CI): 2.8 (1.7–4.5), *p* < 0.0001), and no CR after one course induction (HR (95%CI): 3.9 (1.9–8.0), *p* < 0.0001) were independent poor prognostic factors (Figure 3).

**Table 3:** Univariate and multivariate analysis of OS in the entire cohort.

Variable	Univariate analysis		Multivariate analysis	
	4-year RFS Rate (95%CI)	*p* values*	HR (95%CI)	*p* values
*EVI1* transcript levels		<0.0001		0.0090
*EVI1*-L	57.0% (50.7–62.8%)		2.5 (1.3–5.1)	
*EVI1*-H	82.7% (73.6–88.9%)		1.0	
Age (year)		< 0.0001		0.011
<40	69.6% (63.2–75.1%)		1.0	
≥40	51.9% (42.6–60.4%)		1.8 (1.1–3.0)	
Sex		0.29		
Male	60.9% (53.0–67.9%)			
Female	67.0% (59.8–73.2%)			
WBC (x10^9^/L)		0.0070		
<20	67.4% (61.2–72.8%)			
≥20	54.4% (43.8–63.8%)			
Hb (g/L)		0.34		
<87	64.6% (57.0–71.3%)			
≥87	63.9% (56.6–70.3)			
Plt (x10^9^/L)		0.037		
<67	57.2% (49.2–64.3%)			
≥67	69.3% (62.2–75.3%)			
Cytogenetic categories (*n* = 335)		0.0020		
High-risk karyotype	52.0% (36.0–65.7%)			
Non-high-risk karyotype	71.3% (65.0–76.7%)			
*IKZF1* deletion		0.097		
Positive	65.1% (47.7–77.9%)			
Negative	80.9% (73.7–86.4%)			
Fusion-defined risk group		0.0030		
High risk	47.1% (35.2–58.1%)			
Standard risk	68.1% (62.2–73.2%)			
Treatment methods		< 0.0001		<0.0001
Allo-HSCT	78.6% (72.5–83.5%)		1.0	
Chemotherapy only	45.3% (35.6–54.5%)		2.8 (1.7–4.5)	
Achieving CR after 1-course induction		< 0.0001		<0.0001
Yes	69.4% (63.9–74.2%)		1.0	
No	20.3% (9.6–33.8%)		3.9 (1.9–8.0)	
MRD > 0.01% after first consolidation (*n* = 363)		0.77		
Yes	65.0% (54.7–73.5%)			
No	68.4% (61.5–74.4%)			

*Variables with *p* < 0.05 from the univariate analyses were entered into the multivariate model using Cox proportional hazards models.

### The impact of *EVI1* expression on outcome within the individual fusion gene groups

We first performed survival analyses of patients with or without the fusion gene. *KMT2A* rearrangement and *MEF2D* fusions were significantly related to worse RFS (*p* < 0.0001 and 0.001) and *ZNF384* fusions were significantly related to better RFS (*p* = 0.003), whereas *TCF3::PBX1* and *Ph-like* had no relation to RFS (all *p* = 0.22). Moreover, *KMT2A* rearrangement is significantly related to worse OS (*p* = 0.012), and *ZNF384* fusions are significantly related to better OS (*p* = 0.025).

As shown in Table 1, all patients (100%) with *KMT2A* rearrangement and the vast majority of patients (> 90%) with *TCF3::PBX1* or *MEF2D* fusions were classified as *EVI1*-L if using the cutoff values (2.3%) applied to the whole cohort. Thus, we performed quartile stratification for each fusion gene subgroup and found no significant difference in RFS and OS among quartile groups (all *p* > 0.05). Then, we performed ROC curve analysis using relapse or not as a distinguishing variable to determine the cutoff value for *EVI1* transcript levels within each fusion-defined group. As a result, 0.33, 0.05, 2.0, 0.5, and 0.9% were optimal thresholds for *TCF3::PBX1*, *KMT2A* rearrangement, *ZNF384* fusion, *MEF2D* fusion, and *Ph-like* fusion groups, and patients with *EVI1* levels greater than or equal to the cutoff value and those with levels less than the cutoff value were defined as the *EVI1*-H and *EVI1*-L, respectively. We further evaluated the impact of *EVI1* expression on RFS and OS within the individual group. The RFS of patients in the *EVI1*-L group was or tended to be significantly inferior to that of patients in the *EVI1*-H group within *TCF3::PBX1*, *Ph-like* fusions, *MEF2D* fusions, and *ZNF384* fusions cohorts (*p* = 0.0047, 0.025, 0.032, and 0.07), respectively. The OS of the *EVI1*-L group was significantly poorer than that of the *EVI1*-H group in the *ZNF384* fusions cohort (*p* = 0.012). There was no difference in both RFS and OS between the *EVI1*-L and the *EVI1*-H groups in *KMT2A* rearrangement cohort.

**Figure 4:**
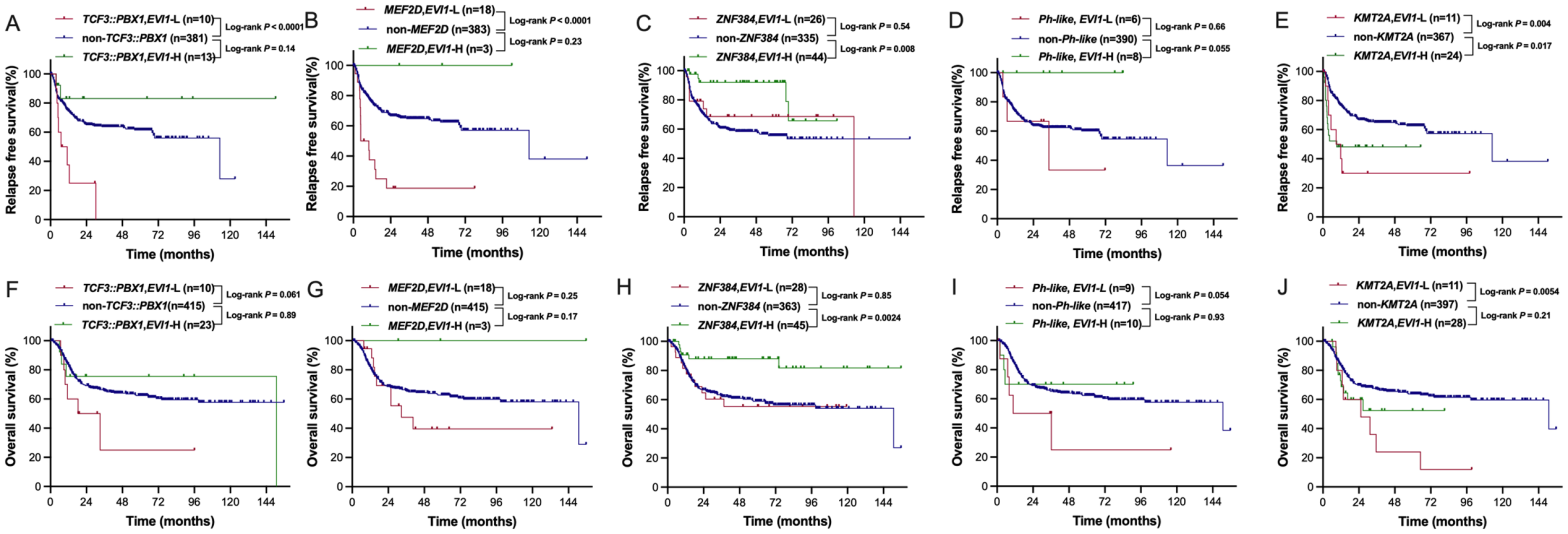
RFS and OS in fusion-defined groups. RFS **(A)** and OS **(F)** in the *TCF3::PBX1* group; RFS **(B)** and OS **(G)** in the *MEF2D* fusions group; RFS **(C)** and OS **(H)** in the *ZNF384* fusions group; RFS **(D)** and OS **(I)** in the *Ph-like* fusions group; RFS **(E)** and OS **(J)** in the *KMT2A* rearrangement group. The optimal *EVI1* expression cutoff was individually determined for each genetic subgroup using receiver operating characteristic (ROC) curve analysis.

We further made comparisons in the whole cohort, in which patients were categorized into the following three groups: *EVI1*-H with the fusion gene, *EVI1*-L with the fusion gene, and without the corresponding fusion gene. As shown in Figure 4, patients in the *EVI1*-H with *TCF3::PBX1* or *MEF2D* fusion group had similar RFS and OS in both cohorts compared to those without the corresponding fusions (*TCF3::PBX1*: *p* = 0.14 and 0.89; *MEF2D*: *p* = 0.23 and 0.17), while patients in the *EVI1*-L with the *TCF3::PBX1* group exhibited a significantly poorer RFS or tended to have significantly poorer OS (*p* < 0.0001 and 0.061) compared to those without *TCF3::PBX1*, and patients in the *EVI1*-L with *MEF2D* fusion group exhibited a significantly poorer RFS and similar OS (*p* < 0.0001 and 0.25) compared to those without *MEF2D* fusion. In *Ph-like* cohort, patients in the *EVI1*-L group had similar RFS and tended to have poorer OS (*p* = 0.66 and 0.054), and those in the *EVI1*-H group tended to have better RFS but similar OS (*p* = 0.055 and = 0.93) than those without the *Ph-like* fusions. In addition, patients in the *EVI1*-L with *ZNF384* fusion group had similar RFS and OS (*p* = 0.54 and 0.85), and *EVI1*-H with *ZNF384* fusion group had significantly better RFS and OS (*p* = 0.008 and 0.0024), compared to those without *ZNF384* fusion. In summary, *EVI1* transcript level serves as a critical prognostic modifier within specific fusion-defined subgroups. High *EVI1* expression identified a lower-risk subgroup within traditionally high-risk categories defined by *TCF3::PBX1*, *MEF2D*, and *Ph-like* fusions. Conversely, low *EVI1* expression defines a higher-risk subgroup within the typically more favorable *ZNF384* fusion group.

## Discussion

In this study, we thoroughly evaluated *EVI1* expression patterns at diagnosis and its prognostic significance in the consecutive 436 adult Ph-negative B-ALL patients. We found that low *EVI1* expression, defined as the lower three quartiles of the quartile distribution, was an independent poor prognostic factor for RFS and OS in the entire cohort. Furthermore, the subgroup analysis showed that low *EVI1* expression was related to poorer RFS within the *TCF3::PBX1*, *MEF2D* fusions and *Ph-like* fusions groups, and related to poorer OS in the *ZNF384* group.

In both adult and pediatric AML, those with high expression of *EVI1* have poor outcomes (9, 10, 29, 30). In contrast, this is not universally observed in ALL. The current findings suggested that the consistent prognostic significance of *EVI1* expression in adults is consistent with pediatric Ph-negative B-ALL patients as we previously reported (18), wherein lower transcript levels being associated with poorer prognosis, despite differences in cutoff values for *EVI1*. Moreover, we found that low *EVI1* expression was an independent poor prognostic factor for both RFS and OS in the current cohort. Abbal et al. assessed *EVI1* gene expression in a cohort of 138 adult Ph-negative B-ALL patients and found no prognostic impact for either low (0.05%) or high (1.65%) *EVI1* expression (14). Similarly, Nabil et al. reported no significant impact of *EVI1* expression on survival in a cohort of 71 adult ALL patients (including both B-ALL and T-ALL) (16). We propose that several methodological factors may account for these discrepant results. First, the criteria for patient inclusion are critical. ALL is a heterogeneous disease, and the inclusion of T-ALL, a subtype with distinct molecular pathogenesis, may obscure subtype-specific prognostic signals. Unlike the above two studies, our analysis was confined to patients with Ph-negative B-ALL, enhancing the homogeneity of our cohort. Second, statistical power is a key consideration. Our study enrolled 436 adult patients, representing the largest cohort to date for this specific investigation, which improves the reliability of the findings. Finally, the methodology for defining *EVI1* expression groups differed substantially. Abbal et al. utilized the 1st and 99th percentiles of a control group, a highly stringent approach that may classify very few patients as having “low” or “high” expression. In contrast, we employed a quartile-based cutoff, defining the lower three quartiles and upper quartile as the low and high expression groups, respectively. This approach provides a more balanced distribution for robust statistical comparison and may be more sensitive for detecting a continuous relationship between expression levels and clinical outcome.

The pathogenic mechanisms of the *EVI1* gene in AML have been partially explored, implicating it in several key signaling pathways. For instance, *EVI1* is known to relieve cellular growth inhibition mediated by transforming growth factor beta (TGF-*β*) (31) and promotes the proliferation of myeloid preleukemic cells by upregulating Spi1 in encoding PU.1. (32). In contrast, the mechanisms of *EVI1* in lymphoid malignancies remain poorly characterized, and only Konantz et al. reported the *in vitro* study results on ALL to date. They showed that the apoptosis rates were significantly elevated after knockdown of *EVI1* expression in ALL cell lines NALM-16 and REH, and found that *EVI1* modulates expression of several apoptosis-related genes (such as *BCL2*, *BCL-x*, *XIAP*, *NOXA*, *PUMA*, *TRAIL-R1*) (17). Our research team is currently conducting in vitro study to investigate the mechanism of *EVI1* expression on B-ALL cells.

In AML, the hallmark of 3q26.2 rearrangement is high *EVI1* expression, primarily driven by the hijacking of the GATA2 distal hematopoietic enhancer (4, 33, 34). In contrast, 3q26.2 rearrangement is exceptionally rare in ALL, having been reported only in isolated cases of secondary ALL (11, 12). Consistent with this finding, our analysis of this Ph-negative B-ALL cohort identified no evidence of 3q26.2 chromosomal abnormalities or cryptic 3q26.2 rearrangements. This absence strongly suggests that the mechanism underlying *EVI1* overexpression in this context is independent of 3q26.2 rearrangement (9, 34, 35).

We observed no significant differences between the low and high *EVI1* expression groups in adult Ph-negative B-ALL patients across multiple clinical parameters, including sex, age, WBC count, and hemoglobin. These findings are consistent with those of other studies. Konantz et al. showed that *EVI1* expression is not associated with age and WBC count in ALL (17), a result corroborated by Abbal et al. in their adult Ph-negative B-ALL cohort (14). In contrast, unlike our research on adult ALL, Steven et al. discovered that *EVI1* expression is age-related and hypothesized that TGF-*β* might be a significant factor influencing-related gene expression in pediatric ALL (13). Moreover, significant differences emerged in the distribution of specific molecular and cytogenetic alterations. The *EVI1* expression groups were distinct in their prevalence of *KMT2A* rearrangements, *TCF3::PBX1* fusions, *ZNF384* fusions, *IKZF1* deletions, and certain cytogenetic categories. This finding, however, diverges from the report by Abbal et al., who did not observe significant associations between *EVI1* expression and *IKZF1* deletions or complex karyotypes (14). We posit that several factors may account for these discrepant results. Key considerations include differences in cohort composition (e.g., the specific distribution of genetic subtypes), statistical power due to varying sample sizes, and potentially the statistical methodologies employed.

Differing from a significant correlation between high *EVI1* expression and *KMT2A* rearrangement established in both adult and pediatric AML patients (9, 10, 13, 17, 36), our observations in adult Ph-negative B-ALL patients indicate an association between *KMT2A* rearrangement and uniformly fairly low *EVI1* expression levels (median, 0.090%, range: 0.0040–0.50%). This finding is consistent with our previous findings in pediatric B-ALL (18) and are further supported by literatures performed by Abbal et al. and Konantz et al., in which a significant downregulation of *EVI1* gene expression were also found in adult Ph-negative ALL patients with the *KMT2A::AFF1*+/ t(4;11) rearrangement (14, 17).

In this study, all patients were tested for not only classical but also new fusion genes, all of which serve as critical indicators for genetic stratification in B-ALL. Currently, risk stratification by fusion gene in B-ALL remains inconsistent. The 2024 NCCN guidelines classify *ZNF384* fusions as high-risk and *TCF3::PBX1* as standard-risk, while the 2024 ELN recommendations categorize them as intermediate-risk and favorable-risk, respectively (19, 20). A multinational RNA-sequencing study of 1,223 B-ALL cases found that *ZNF384* fusions confer an intermediate-risk and *TCF3::PBX1* a high-risk profile in adult B-ALL patients (37). In line with this study, our center’s consecutive decade-long cohort study identified *ZNF384* fusions as standard-risk and *TCF3::PBX1* as high-risk (25, 28). Similarly, a Japanese study reported the favorable prognosis of *ZNF384* in adult B-ALL patients (38). Regarding the *Ph-like* subtype in our cohort, our previous study showed that it did not have inferior RFS but poor OS, which was caused by low 1-course CR rate (28). In addition, the *Ph-like* subtype sample size is limited (*n* = 19), which is consistent with data from Taiwan but lower than those from Western populations (19, 20, 39). These discrepancies may stem from ethnic variations and differences in treatment strategies. In particular, the use of allo-HSCT may significantly affect the prognosis of adult Ph-negative B-ALL.

To our knowledge, no prior study has integrated *EVI1* transcript levels with both classical and new fusion genes to assess their combined prognostic significance. In the present cohort, the highest level of *EVI1* expression was observed in *ZNF384* fusions group. Given the favorable prognosis associated with *ZNF384* fusions in our previous reports (25, 28), this may be one reason for the good prognosis of the high *EVI1* expression. In order to clarify the prognostic role of *EVI1* transcript levels, we evaluated the prognostic significance of *EVI1* within each fusion-defined subgroup. We found that low *EVI1* expression is related to poorer RFS in the *TCF3::PBX1*, *MEF2D* fusion and *Ph-like* fusion groups, with poorer OS—and tended to related to poorer RFS—in the *ZNF384* group. No significant difference in both RFS and OS between the *EVI1*-L and the *EVI1*-H subgroups in *KMT2A* rearrangement cohort was observed.

Furthermore, we attempted to combine *EVI1* expression with a fusion gene for further risk stratification of the entire cohort. Among patients with high-risk fusion (*TCF3::PBX1*, *MEF2D,* and *Ph-like*), those with high *EVI1* expression exhibited favorable outcomes comparable to those without such fusions. Conversely, among patients with *ZNF384* fusions, low *EVI1* expression identified a subgroup with outcomes similarly unfavorable to those of high-risk fusion carriers. It illustrated that *EVI1* expression may improve fusion-defined risk stratification in adult Ph-negative B-ALL.

In conclusion, our study demonstrates that *EVI1* transcript levels at diagnosis are highly variable and exhibit a distinct association with specific fusion gene subtypes in adult Ph-negative B-ALL. Critically, low *EVI1* expression served as a powerful, independent predictor of adverse outcomes, not only in the overall cohort but also within key molecular subgroups, including those with *TCF3::PBX1*, *Ph-like*, *MEF2D*, and *ZNF384* fusions. Notably, the integration of *EVI1* expression with fusion gene status refined risk stratification, effectively identifying standard-risk patients within traditionally high-risk fusion groups and high-risk patients within otherwise favorable-risk groups. These findings underscore the clinical utility of incorporating *EVI1* transcript quantification into the initial diagnostic workup of adult Ph-negative B-ALL. We acknowledge the limitations inherent in our retrospective study design and the heterogeneity in treatment protocols. Therefore, prospective validation in independent, multi-center cohorts is essential to confirm the generalizability of our observations and to further elucidate the prognostic and potential therapeutic implications of *EVI1* expression in the context of modern, molecularly defined ALL therapy.

## Data Availability

The original contributions presented in the study are included in the article/Supplementary material, further inquiries can be directed to the corresponding author.
