# Supplementary material for: Low EVI1 expression at diagnosis identifies a high-risk subgroup in adult Ph-negative B-cell acute lymphoblastic leukemia
Source: Front Med (Lausanne). 2026 Jan 13;12:1701539. doi: 10.3389/fmed.2025.1701539 (PMC12834734; doi:10.3389/fmed.2025.1701539)
Supplement: Supplementary file 1 [file Table_1.DOCX]

| Subgroup | N,% | *EVI1* transcript levels  (median,range) |
| --- | --- | --- |
| *TCF3::PBX1*/t(1;19) | 23 (5.3%) | 0.41% (0.013 - 14.7%) |
| *KMT2A* rearrangement/t(4;11) | 39 (8.9%) | 0.090% (0.0040 - 0.50%) |
| *KMT2A::AFF1* | 35 |  |
| *KMT2A::MLLT1* | 3 |  |
| *KMT2A::EPS15* | 1 |  |
| *ZNF384* fusion | 73 (16.7%) | 5.8% (0.11 - 70.6%) |
| *EP300::ZNF384* | 56 |  |
| *CREBBP::ZNF384* | 8 |  |
| *TAF15::ZNF384* | 4 |  |
| *TCF3::ZNF384* | 3 |  |
| *EWSR1::ZNF384* | 2 |  |
| *MEF2D* fusion | 21 (4.8%) | 0.14% (0.034 - 4.3%) |
| *MEF2D::BCL9* | 11 |  |
| *MEF2D::HNRNPUL1* | 8 |  |
| *MEF2D::DAZAP1* | 1 |  |
| *MEF2D::FOXJ2* | 1 |  |
| *Ph-like* fusion | 19 (4.3%) | 1.2% (0.045 - 72.9%) |
| *P2RY8::CRLF2* | 6 |  |
| *EBF1::PDGFRB* | 3 |  |
| *RCSD1::ABL2* | 3 |  |
| *NUP214::ABL1* | 2 |  |
| *TEL::ABL1* | 1 |  |
| *EBF1::JAK2* | 1 |  |
| *PCM1::JAK2* | 1 |  |
| *PAX5::JAK2* | 1 |  |
| *BCR::FGFR1* | 1 |  |

Table S1: The molecular characteristics of the cohort and *EVI1* expression per subgroup.
