# Supplementary figures and images for: Low EVI1 expression at diagnosis identifies a high-risk subgroup in adult Ph-negative B-cell acute lymphoblastic leukemia
Source: Front Med (Lausanne). 2026 Jan 13;12:1701539. doi: 10.3389/fmed.2025.1701539 (PMC12834734; doi:10.3389/fmed.2025.1701539)

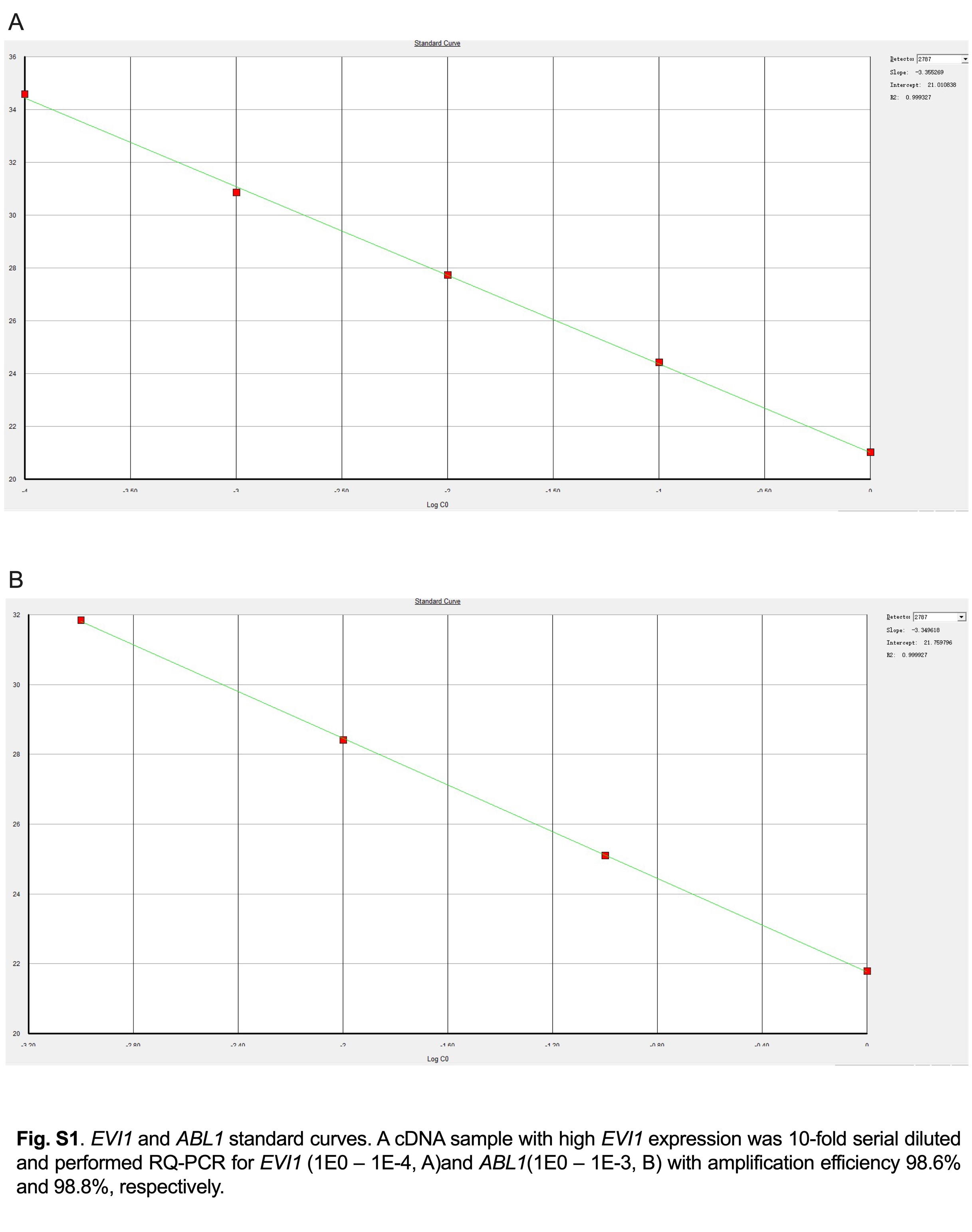

Supplement: Supplementary file 2 [file Image_1.tiff]

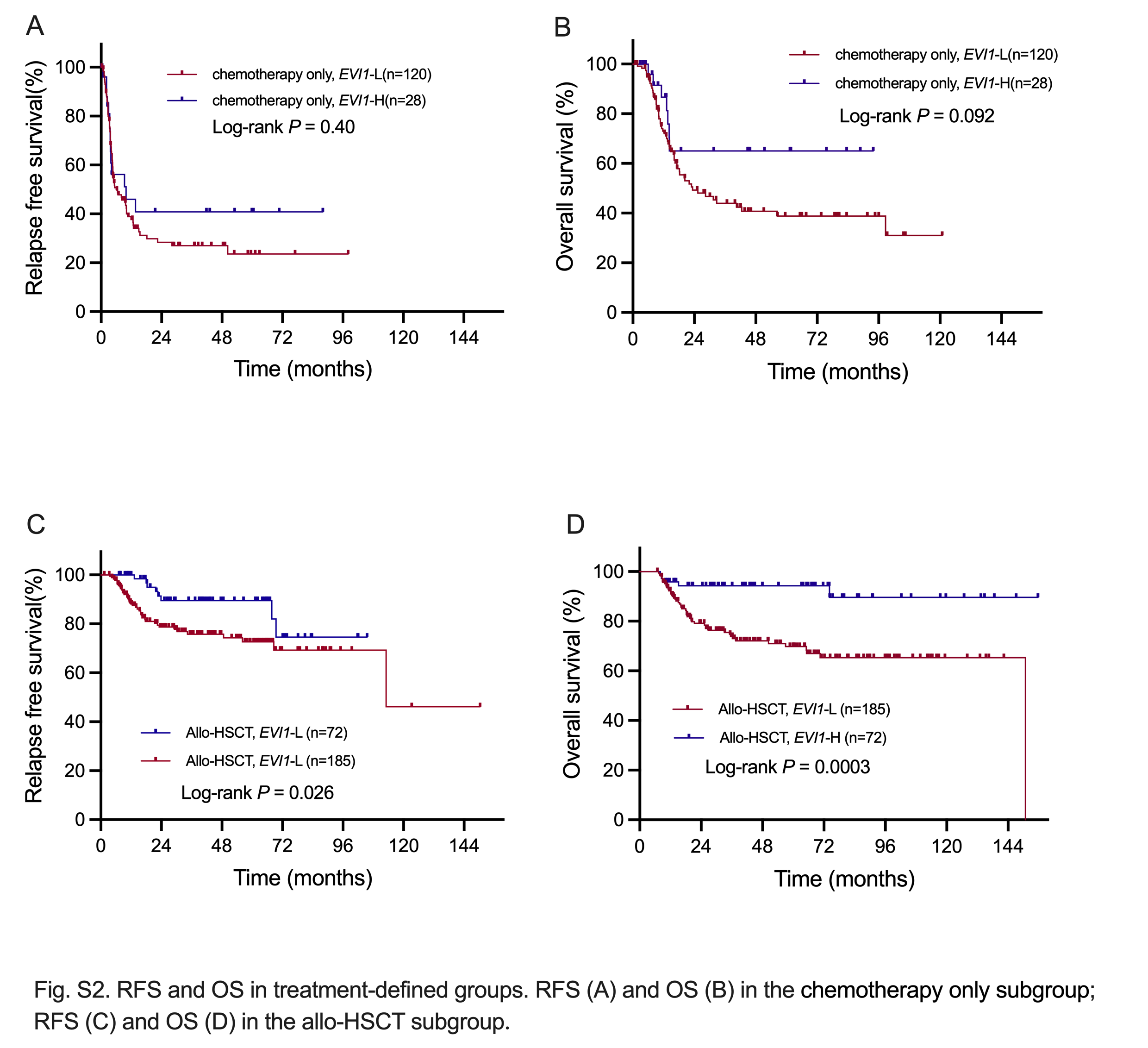

Supplement: Supplementary file 3 [file Image_2.tiff]
